# Classification of protein sequences by means of irredundant patterns

**DOI:** 10.1186/1471-2105-11-S1-S16

**Published:** 2010-01-18

**Authors:** Matteo Comin, Davide Verzotto

**Affiliations:** 1 Department of Information Engineering, University of Padova, Italy

## Abstract

**Background:**

The classification of protein sequences using string algorithms provides valuable insights for protein function prediction. Several methods, based on a variety of different patterns, have been previously proposed. Almost all string-based approaches discover patterns that are not "independent, " and therefore the associated scores overcount, a multiple number of times, the contribution of patterns that cover the same region of a sequence.

**Results:**

In this paper we use a class of patterns, called irredundant, that is specifically designed to address this issue. Loosely speaking the set of irredundant patterns is the smallest class of "independent" patterns that can describe all common patterns in two sequences, thus they avoid overcounting. We present a novel discriminative method, called Irredundant Class, based on the statistics of irredundant patterns combined with the power of support vector machines.

**Conclusion:**

Tests on benchmark data show that Irredundant Class outperforms most of the string algorithms previously proposed, and it achieves results as good as current state-of-the-art methods. Moreover the footprints of the most discriminative irredundant patterns can be used to guide the identification of functional regions in protein sequences.

## Background

The increasing availability of biological sequences, from proteins to entire genomes, poses the need for the automatic analysis and classification of such a huge collection of data. Alignment methods and pattern discovery techniques have been used, for quite some time, to attach various problems emerging in the field of computational biology. Unfortunately most of these methods do not scale well with the lengths of the biological sequences under examination and therefore they are unpractical for genome-wide applications. To overcome this recent obstacle a number of techniques, which do not relay on alignment, have been conceived; these methods are also called alignment-free methods (see [1] for a comprehensive review). Although several alignment methods have been proposed over the years, the development of tools to classify and digest entire genome sequences is still in its infancy.

In this paper we address the classification and analysis of biological sequences without resorting to alignment. We present a method for the classification of protein sequences by means of *irredundant patterns*. Our method achieves results as good as the current state-of-the-art methods. We show also that the most discriminative irredundant patterns, which are used to distinguish different protein families, provide valuable information on the consensus pattern shared by a particular protein family.

### Related works

The protein classification problem can also be treated as a string classification problem. Historically this problem has been studied in the field of text documents classification. Unfortunately most of these approaches, developed for a different kind of strings, fail when applied to biological sequences. The main reasons of this failure are the different nature of biological sequences, particularly rich of regularities known as patterns, and because of their lengths difficult to digest for a general purpose application.

Thus a number of methods have been studied for protein classification based on primary structure. The main distinction is between generative methods against discriminative methods. The former class includes methods such as protein family profiles [2], hidden Markov models (HMMs) [3-5], and PSI-BLAST [6]. These methods try to derive a model for a set of proteins and then check whether a candidate protein fits the model or not. Unlike generative methods, discriminative methods such as [7-14] are supervised approaches focused on finding which sequences (including negative examples) can describe a set of proteins despite of another set. This class makes extensive use of support vector machines (SVMs, [15]) based on features of proteins.

Recent results [8,10] suggest that the best-performing methods are discriminative string algorithms, sometimes called string kernels. The string-based learning algorithm extracts information from the sequences and computes either a feature vector for each sequence or directly a matrix with scores between pairs of sequences. Then sequences are seen as a set of labeled examples (positive if they are in the family and negative otherwise) and an SVM attempts to learn a decision boundary between the different classes. The first string kernel, called Fisher kernel [7], uses an HMM to provide the necessary means of converting proteins into fixed-length vectors. The vector summarizes how different the given sequence is from a typical member of the given protein family. In contrast in the Pairwise method [8] the feature vector, corresponding to a protein, is formed by all the *E*-values of the Smith-Waterman score (or BLAST score) between the sequence analyzed and each of the training sequences. Other methods, like Spectrum and Mismatch [9,10], use as protein features the set of all possible substrings of amino acids of fixed length *k *(*k*-mer). If two sequences contain many of the same *k*-mers, their inner product under the *k*-Spectrum kernel will be large. Equivalently, the Mismatch kernel computes a large inner product between two sequences if these sequences contain many *k*-mers that differ by at most *m *mismatches. Then, the SVM-I-sites method [12] encodes into feature vectors the protein's three-dimensional structure, instead of using sequence similarity; conversely the eMOTIF database method [16] defines the kernel in terms of the sequence motifs that appear in a pair of sequences. These motifs were previously extracted from the eBLOCKs database using the eMOTIF-maker algorithm that derives patterns from sequence alignments [17,18].

More recently, the Local Alignment method [11] mimics the behavior of the Smith-Waterman score to build a family of valid kernels. Following the work of [19] they defined a kernel to detect all local alignment between strings by convolving simpler kernels, and hence comparing, in a simple way, strings of different lengths which share common parts. The Profile-based Mismatch method [14] uses probabilistic profiles, such as those produced by the PSI-BLAST algorithm, to define a kernel established on the position-dependent mutation neighborhoods for inexact matching of *k*-mers in the data (like the Mismatch method). Whereas in the Profile-based Direct method [13] kernel functions are constructed by combining sequence profiles with different approaches for determining the similarity between pairs of protein sequences. However, this kernel makes an extensive use of hyperparameters that increases the risk of overfitting when no dedicated validation data set is used. Finally, in the Word Correlation Matrices method [20] the kernel is defined by average pairwise *k*-mer (or word) similarity between two sequences (similarly to the Spectrum kernel). The consequent analysis of discriminative words allows also the identification of characteristic regions in biological sequences.

In general, all pattern-based methods operate, directly or indirectly, two distinct steps: first extract patterns from sequences, then use this set of patterns as features for an automatic classification tool, e.g. SVMs.

Remarkably almost all string algorithms consider patterns that are not independent, and therefore the associated scores are obtained using a set of redundant features. In this paper we want to stress the idea that if the learning process has to deal with a set of redundant features, the resulting score is overcounting, a multiple number of times, the contribution of patterns that cover the same region of a sequence. Our conjecture is that the overcounting, due to redundant patterns, might mislead the classification. In the case of pattern-based method we can define if two patterns are independent through the resort of the notion of *irredundancy*. The class of *irredundant patterns *is specifically designed to address this issue and it was previously studied in a number of papers [21-24]. In [25] we applied a similar technique for the detection of transcription factor binding sites and we showed that the use of irredundancy is useful since redundant patterns can be filtered out without leading to overcounts.

In the next sections we will show that most approaches are using patterns, of various forms, that are redundant. We suppose that a set of irredundant patterns, and consequently a set of independent features, can improve the automatic learning and classification of sequences. Here we present a novel discriminative pairwise method for the classification of protein sequences, called *Irredundant Class*. *Irredundant Class *is based on the statistics of irredundant patterns between two sequences, combined with the discriminative power of SVMs. We selected for comparison some of the string-based learning approaches presented above, including the best-performing methods in the protein classification and remote homology detection [7-11, 20]. Our method outperforms most of the previous approaches, and it achieves results as good as current state-of-the-art methods. Moreover, given a protein family, we study the set of most discriminative irredundant patterns and their distribution. We found that the location of the functional consensus pattern for a family, as reported by PROSITE [26], can be identified by the irredundant patterns footprint.

## Methods

### Irredundant Class

Our method is based on the extraction of irredundant patterns that are common to two sequences. We use the idea of irredundant patterns, to avoid overcounting of patterns that cover the same region multiple times on a sequence. In this section we present the class of irredundant patterns, along with some properties.

Recently the notion of irredundancy was studied for the case of a single sequence [21,22], in this paper we extend the notion of irredundancy to the case of two sequences. In particular, our approach is substantially inspired by [22], and we refer the reader for a more comprehensive treatment on the original notion. To keep the paper self-contained, we report here the basic concepts, although most of them are only slightly adapted.

Let *s *= *σ*_1_*σ*_2 _... *σ*_*n *_be a sequence of length |*s*| = *n *over an alphabet Σ. A character from Σ, say *σ*, is called a *solid *character, while a "don't care" character '.' represents any character. A *pattern *is a string over Σ·(Σ ∪{.})*·Σ, thus containing at least two solid characters. For instance, *p *= *d.dg.g.i...e *is a pattern that occurs at locations 1 and 15 in the sequence *s *= *dadgggdistketvdedgsgtidfee*.

To give an idea of the notion of irredundancy applied to two sequences *s*_1 _and *s*_2_, let us consider *s*_1 _= *abababab *and *s*_2 _= *babababa*, and the two patterns *p*_1 _= *abababa *and *p*_2 _= *ababa *that are contained in both the sequences. We can note that the existence of *p*_1 _in both *s*_1 _and *s*_2 _affects the existence of *p*_2 _in all locations in which *p*_2 _appears. By simply deleting the last *ba *from *p*_1 _or right shifting twice *p*_2 _along *p*_1 _we can obtain *p*_2_. Loosely speaking the two patterns *p*_1 _and *p*_2 _are not independent, or equivalently they are not irredundant. Intuitively, a pattern is irredundant if it cannot be deduced, along with its location list, by some other patterns. Consequently any redundant pattern can be derived from the set of all irredundant patterns without knowing the original sequences, thus it is not informative. We want to discard all redundant common patterns as non-informative for the learning process.

**Definition 1**. *(Common pattern p, Location list *ℒ_*p*_) *Let s *_1_*and s*_2 _*be two sequences on *Σ. *A string p on *Σ ∪ {.} *is a common pattern with location list *ℒ_*p *_= (*l*_1_, *l*_2_, ..., *l*_*q*_) *if all of the following hold. (i) *|*p*| ≥ 2, *(ii) p*[1], *p*[|*p*|] ∈ Σ, *(iii) p occurs both on s*_1 _*and s*_2_, *and (iv) there exists no location l *∉ ℒ_*p *_*such that p occurs at l either on s*_1 _*or s*_2 _*(the location list is of maximal size)*.

Clearly, in the two sequences context, a common pattern occurs at least twice, one per sequence. Extending the notation of location list we can then denote by (ℒ_*p*_+ *i*), 0 ≤ *i *≤ |*p*| - 1, *all the locations in *ℒ_*p *_shifted to the right by *i *positions.

**Definition 2**. *(p*_1 _≼ *p*_2_) *For characters σ*_1 _*and σ*_2 _*we write that σ*_1 _≼ *σ*_2 _*if and only if σ*_1 _*is a don't care or σ*_1 _= *σ*_2_. *Given two patterns p*_1 _*and p*_2_, *with *|*p*_1_| ≤ |*p*_2_|, *p*_1 _≼ *p*_2 _*holds if p*_1_|*j*| ≼ *p*_2_[*j *+ *d*] *for all *1 ≤ *j *≤ |*p*_1_|, *with *0 ≤ *d *≤ |*p*_2_| - |*p*_1_|.

We also say in this case that *p*_1 _is a *subpattern *of *p*_2 _(or *p*_1 _occurs in *p*_2_), and that *p*_2 _implies or extends *p*_1_.

**Definition 3**. *(Coverage) Given two patterns p*_1 _*and p*_2 _*we say that the occurrence at j of p*_2 _*is covered by p*_1 _*(or by a subpattern of p*_1 _) *if p*_2 _≼ *p*_1 _*and j *∈ ( + *i*) *for an integer *0 ≤ *i *≤ |*p*_1_| - |*p*_2_|.

For instance, the pattern *p*_2 _= *ababa *with location list  over *s*_1 _= *abababab *and *s*_2 _= *babababa*, where  denotes the occurrence at location *j *in the sequence *s*_*h*_, is covered at position  by *p*_1 _= *abababa *with  and *i *= 2. Note that  because *p*_2 _is a subpattern of *p*_1 _obtained by deleting the last *ba *from *p*_1 _(i.e., the shift integer *i *is 0), and that  because *p*_2 _occurs at location 2 in *p*_1_. Another example with don't cares is the following, *p*_3 _= *a.a.a *with  over *s*_3 _= *aabababab *and *s*_4 _= *babacacac *is covered at all the positions by *p*_4 _= *aba.a.a *with .

**Remark 1**. *Let s*_1 _*and s*_2 _*be two sequences, and p*_1 _*and p*_2 _*be two common patterns with p*_1 _≼ *p*_2_. *Then, by definition p*_2 _*must cover at least an occurrence of p*_1 _*per sequence*.

**Definition 4**. *(Maximal common pattern) Let **be the set of common patterns of the sequences s*_1 _*and s*_2_. *A pattern **is maximal in composition if and only if there exists no *, *l *≠ *i, with p_i _≼ p_l _and . A pattern p_i _maximal in composition is also maximal in length if and only if there exists no pattern *, *j *≠ *i, such that p*_*i *_≼ *p*_*j *_*and *. *A maximal common pattern is a pattern that is maximal both in composition and length*.

Requiring maximality in composition and length limits the number of common patterns that may be usefully extracted and accounted for in two sequences. However, the notion of maximality alone does not suffice to bound the number of such patterns. It can be shown that there are sequences that have an unusually large number of maximal common patterns without conveying extra information about the input. A maximal common pattern *p *is *irredundant *if *p *and the list ℒ_*p *_of its occurrences cannot be deduced by the union of a number of lists of subpatterns of other maximal common patterns. Conversely, we call a common pattern *p redundant *if *p *(and its location list ℒ_*p*_) can be deduced from the other common patterns without knowing the input sequences. More formally:

**Definition 5**. *(Redundant/Irredundant common pattern) A maximal common pattern p, with location list ℒ_p_, is redundant if there exist maximal common subpatterns p_i_*, 1 ≤ *i *≤*k, such that **(i.e., every occurrence of p on s*_1 _*and s*_2 _*is already covered by some maximal common patterns). A maximal common pattern that is not redundant is called irredundant common pattern*.

Since the redundant common patterns bring no extra information about the two sequences, the set of independent patterns is precisely the class of irredundant common patterns. For instance, we consider the sequences *s*_1 _= *abababab *and *s*_2 _= *babababa *of length 8. Then the list of all irredundant common patterns is the following. *p*_1 _= *abababa *with , *p*_2 _= *bababab *with . The other redundant maximal common patterns are. *p*_3 _= *ababab *with , *p*_4 _= *bababa *with , *p*_5 _= *ababa *with , *p*_6 _= *babab *with , *p*_7 _= *abab *with , *p*_8 _= *baba *with , *p*_9 _= *aba *with , *p*_10 _= *bab *with , *p*_11 _= *ab *with , *p*_12 _= *ba *with .

It is easy to check that *p*_1 _and *p*_2 _cannot be deduced by other common patterns, whereas *p*_5 _along with its location list can be simply deduced by *p*_1_, and *p*_7 _can be derived from the union of the occurrence lists of some subpatterns of *p*_1 _and *p*_2_. We want to emphasize that if the two sequences are identical there is only one irredundant common pattern, the sequence itself. This difference with the single sequence approach is due to a peculiarity of the original notion, in which the sequence itself is not considered as a valid pattern.

**Definition 6**. *(Consensus, Meet) The consensus of two sequences s*_1 _*and s*_2 _*is obtained by matching the characters corresponding to the same positions of the sequences, and inserting a don't care in case of mismatch. Deleting all leading and trailing don't cares from the consensus yields the meet of s*_1 _*and s*_2_.

For instance, the consensus of the sequences *aaaaab *and *baaaaa *is *.aaaa*., while their meet is *aaaa*. Note that a meet is a common pattern between two sequences.

Let now *s *= *σ*_1_*σ*_2 _... *σ*_*n *_be a sequence of *n *characters over an alphabet Σ. We use  to denote the suffix *σ_j_σ_j_*_+1_... *σ*_*n*_of *s*; *s*^(*j*) ^the sequence where the location *j *appears; and  the location list of *p *on *s*. Clearly ; and *j *∈ ℒ_*p*_if and only if .

In the following we will briefly present the most important properties of the irredundant common patterns. Those properties are specular with respect to the single sequence approach, as in [22].

**Lemma 1**. *Let p be a common pattern on s*_1 _*and s*_2_. *Then, p is irredundant if and only if there exists **such that the meet of s*^(min{*j, k*})^*and **is p for all *, *where s*_*h *_*is the other sequence with respect to s*^(*j*)^.

*Proof*. We can prove this Lemma using the Lemmas 4 and 5 described in [22]. They prove that a pattern *p' *is irredundant, relatively to the approach with a single sequence *s*, if and only if there exists an occurrence *j' *of *p' *in *s *such that the meet of *s *and  is *p' *for all *k' *in . Following this work, we have that a pattern *p' *is irredundant if and only if at least one of its occurrences in *s *is not covered by other patterns. Similarly a common pattern *p *is irredundant, in the two sequences approach, if and only if it has at least an occurrence *j *in *s*_1_, or in *s*_2_, that is not covered by other maximal common patterns. Now assume, w.l.o.g., that *j *∈ *s*_1_, and therefore *s*_*h *_is *s*_2_. To make the statement of the Lemma valid we have to examine the case in which a common pattern *p'' *covers together the occurrence *j *of *p *in *s*_1 _and another occurrence *l *of *p *in *s*_1 _(i.e., the same sequence in which *j *appears). Note that *p'' *covers some occurrences of *p *if *p *≼ *p"*; hence, by definition, *p" *must cover also an occurrence of *p *in *s*_2 _(as seen in Remark 1). This contradicts the assumptions in which we have to obtain *p *whenever we intersect the sequences in correspondence of two particular occurrences of *p*: *j *in *s*_1 _and a  in *s*_2_.

By the previous facts and the proofs in [22], we can conclude that an irredundant common pattern must have at least an occurrence *j *in one of the two sequences such that the second part of the Lemma holds, and vice versa.   □

To clarify the meaning of this lemma, we refer the reader to the general example that follows the algorithm.

**Theorem 1**. *Every irredundant common pattern over two sequences s*_1 _*and s*_2 _*is the meet of a sequence and a suffix of another one*.

*Proof*. From Lemma 1, an irredundant common pattern *p *(that occurs certainly in both the sequences) must appear at least in the meet of a sequence and a suffix of the other one.   □

An immediate consequence of Theorem 1 is a linear bound for the cardinality of the set of irredundant common patterns. Thus

**Theorem 2**. *The number of irredundant common patterns over two sequences s*_1 _*and s*_2 _*of length, respectively, m and n is O*(*m *+ *n*).

*Proof*. From Theorem 1 we have at most *m *+ *n *- 1 irredundant common patterns, and so *O*(*m *+ *n*).   □

With its underlying convolutory structure, Lemma 1 suggests an immediate way for the extraction of irredundant common patterns from sequences and arrays, using available pattern matching with or without FFT [22].

### The algorithm

The discovery of all irredundant common patterns over two sequences *s*_1 _and *s*_2 _is derived from Lemma 1. In this context we are interested in a proof of concept, because the aim is to verify the effectiveness of our method in the classification of protein sequences. The algorithm complexity is dominated by the most computationally intensive operation, step (b), which is the global searching of all occurrences of patterns in the sequences. We implemented this step by means of a naive string searching algorithm with don't cares that accounts for *O*((*m *+ *n*)^3^) time, where *m *and *n *are the sizes of *s*_1 _and *s*_2_. The complete description of the algorithm follows.

Input: two sequences *s*_1 _and *s*_2_, where |*s*_1_| = *m *and |*s*_2_| = *n*.

Output: the set of all irredundant common patterns over *s*_1 _and *s*_2_.

1. Compute the *m *+ *n *-1 meets between *s*_1 _or *s*_2 _and a suffix of the other sequence; then discard patterns of length < 2.

2. For each meet *p*:

(a) for each occurrence *j *found in the previous step, called exposed occurrence, increment a counter, *I*_1_[*j*] or *I*_2_[*j*], depending on the sequence in which *j *appears;

(b) perform a string search over *s*_1 _and *s*_2 _to find the number of occurrences of *p *in *s*_1 _and *s*_2_, called respectively *q*_1 _and *q*_2_;

(c) check if the meet *p *is irredundant (see Lemma 1) by finding at least an exposed occurrence *j *of *p *in *s*^(*j*) ^that has a counter value equal to the number of occurrences of *p *in the other sequence (with respect to *s*^(*j*)^). Equivalently, find if there exists an occurrence *j *in *s*_1 _such that *I*_1_[*j*] = *q*_2 _or an occurrence *j *in *s*_2 _such that *I*_2_[*j*] = *q*_1_.

**Example**. Consider the sequences *s*_1 _= *aabababab *and *s*_2 _= *babacacac *of length 9. One of the meets computed by the algorithm is the meet of  and  that is equivalent to compute the meet of  and . Finally, it can be expressed as the meet of *s*_1 _and , which is actually *p *= *a.a.a *(see Table 1).

**Table 1:** Example of meet between a sequence and a suffix of the other sequence.

position*j*	1	2	3	4	5	6	7	8	9		
*s*_2_	*b*	*a*	*b*	*a*	*c*	*a*	*c*	*a*	*c*		
*s*_1_			*a*	*a*	*b*	*a*	*b*	*a*	*b*	*a*	*b*
position *j*			1	2	3	4	5	6	7	8	9
*p*				***a***	.	***a***	.	***a***			

Meet between s_1_ and  with sequences s_1_ = aabababab and s_2_ = babacacac of length 9.

The only exposed occurrences of the pattern *p *are  and  (given by the meet between positions  and ) thus *I*_1 _[2] = 1 and *I*_2 _[4] = 1. Accordingly Table 2 shows the counters, *I*_1 _and *I*_2_, of *p *for each position of *s*_1 _and *s*_2_.

**Table 2:** Example of counters *I*_1 _and *I*_2 _of a meet.

position *j*	1	2	3	4	5	6	7	8	9
*I*_1_[*j*]	0	1	0	0	0	0	0	0	0
position *j*	1	2	3	4	5	6	7	8	9
*I*_2_[*j*]	0	0	0	1	0	0	0	0	0

Counters I_1_ and I_2_ of the pattern p = a.a.a (that is the meet between s_1_ and ) for each position of s_1_ and s_2_ with sequences s_1_ = aabababab and s_2_ = babacacac of length 9.

We note that  and that . Then step (b) performs a string search of *p *over *s*_1 _and *s*_2_. We obtain that  is  with cardinality *q*_1 _= 2, and that  is  with cardinality *q*_2 _= 2. Since *z*_1 _<*q*_2 _and *z*_2 _<*q*_1 _we can conclude by Lemma 1 that *p *is redundant.

### Scoring irredundant patterns

First of all we have to empirically motivate our choice for irredundant patterns rather than considering all maximal patterns. Table 3 shows the number of irredundant against maximal common patterns for 10 pairs of protein sequences used in the final experiments. Results indicate that the number of irredundant patterns tends to be an order of magnitude lower than maximal patterns for not-so-short sequences, and that the latter can be exponential in the size of sequences; thus we avoid the overcount of many sequence regions. Moreover maximal patterns can be prohibitive to extract for some long sequences. Whereas we have already proved that the number of irredundant patterns is at most linear in the size of sequences. Once a set  of irredundant common patterns over two protein sequences *s*_1 _and *s*_2 _is acquired, we compute a scoring function based on the frequency of patterns and on the properties of amino acid compositions. Here we report the general form of the scoring function:

**Table 3:** Irredundant vs. maximal patterns.

No.	*s*_1_	*s*_2_	|*s*_1_| = *m*	|*s*_2_| = *m*	*m *+ *n*	Maximals	Irredundants	% Of irredundants
1.	1alo	1bjt	597	760	1357	≫16697	1256	≪7.5
2.	1qax	1cxp	316	466	782	8397	682	8.1
3.	1gai	1nmt	472	227	699	7037	612	8.7
4.	1cvu	1lgr	511	368	879	9014	787	8.7
5.	1gpe	1yrg	392	343	735	6853	653	9.5
6.	1qqj	3pcc	415	236	651	5090	566	11.1
7.	1bxk	1ofg	352	220	572	3549	489	13.8
8.	1ebf	2nac	169	188	357	1126	277	24.6
9.	1a03	1mho	90	88	178	257	108	42.0
10.	1gpt	1ayj	47	50	97	64	45	70.3

Number of irredundant and maximal common patterns over 10 pairs of protein sequences taken from experiments in Table 4. Rows are sorted according to the percentage of irredundants over the total number of maximal patterns.

where *F*_*p *_is defined as the number of occurrences of the pattern *p *in *s*_1 _and *s*_2_, and *E*[*F*_*p*_] is the expected value of *F*_*p*_. To compute the expected value of *F*_*p *_we assume that the sequences are drawn from an i.i.d. process. The probability of a pattern *p *is simply the product of the probabilities of its symbols *a*_*i *_∈ *p*. If *a*_*i *_is a solid character we compute its probability using the BLOSUM62 substitution matrix [27]; whereas the probability of a don't care is fixed to 1. Since we have assumed that the sequences come from an i.i.d. process, the expected number of occurrences of the pattern *p *in *s*_1 _and *s*_2 _is:

where *m*, *n*, and |*p*| are, respectively, the lengths of the two sequences and the pattern *p*. Given a set of *N *sequences the input for the SVM learning process is the matrix of scores, i.e. *Score*(*s*_*i*_, *s*_*j*_) with 1 ≤ *i*, *j *≤ *N*. The result of this process is a learning distance function that can be treated as an indefinite kernel. When applying SVMs with this kernel, we therefore have to resort to one of the workarounds discussed in [28]. In particular, in case of weak non-positivity of the learning function, we force the optimizer to stop after a maximum number of iterations. Despite these manageable problems, we successfully applied the matrix of scores as a kernel matrix in SVMs, and we retain for future work the task of bridging the gap in the non-positivity of the learning function.

## Results and discussion

We assessed the effectiveness of our method on the classification of protein families into superfamilies. Recent works were compared against the dataset presented in [8]. This dataset is based on the Structural Classification Of Proteins (SCOP, [29]) version 1.53, removing similar sequences with an *E*-value threshold of 10^-25^. The data consist of 4352 sequences grouped into 54 families and 23 superfamilies selected by Liao and Noble. For each family, proteins within the family are considered positive test examples, and proteins within the superfamily but outside the family are considered positive training examples; negative examples are chosen outside the fold, and were randomly split into training and test sets in the same ratio as the positive examples. Therefore this assessment consists of 54 experiments, each corresponding to a target family having at least 10 positive training examples and 5 positive test examples, and no sequence homologies known a priori. In these experiments there is usually an unbalanced number of negative sequences with respect to the number of positive sequences, as illustrated in Table 4. In short, the task consists on classifying target families of sequences into superfamilies of SCOP using an SVM.

**Table 4:** Experiments of Liao and Noble.

		*Training*		*Test*				*Training*		*Test*	
No.	Target family	Pos.	Neg.	Pos.	Neg.	No.	Target family	Pos.	Neg.	Pos.	Neg.
0	7.3.5.2	12	2330	9	1746	27	7.3.10.1	11	423	95	3653
1	2.56.1.2	11	2509	8	1824	28	3.32.1.11	46	3880	5	421
2	3.1.8.1	19	3002	8	1263	29	3.32.1.13	43	3627	8	674
3	3.1.8.3	17	2686	10	1579	30	7.3.6.1	33	3203	9	873
4	1.27.1.1	12	2890	6	1444	31	7.3.6.2	16	1553	26	2523
5	1.27.1.2	10	2408	8	1926	32	7.3.6.4	37	3591	5	485
6	3.42.1.1	29	3208	10	1105	33	2.38.4.1	30	3682	5	613
7	1.45.1.2	33	3650	6	663	34	2.1.1.1	90	3102	31	1068
8	1.4.1.1	26	2256	23	1994	35	2.1.1.2	99	3412	22	758
9	2.9.1.2	17	2370	14	1951	36	3.32.1.1	42	3542	9	759
10	1.4.1.2	41	3557	8	693	37	2.38.4.3	24	2946	11	1349
11	2.9.1.3	26	3625	5	696	38	2.1.1.3	113	3895	8	275
12	1.4.1.3	40	3470	9	780	39	2.1.1.4	88	3033	33	1137
13	2.44.1.2	11	307	140	3894	40	2.38.4.5	26	3191	9	1104
14	2.9.1.4	21	2928	10	1393	41	2.1.1.5	94	3240	27	930
15	3.42.1.5	26	2876	13	1437	42	7.39.1.2	20	3204	7	1121
16	3.2.1.2	37	3002	16	1297	43	2.52.1.2	12	3060	5	1275
17	3.42.1.8	34	3761	5	552	44	7.39.1.3	13	2083	14	2242
18	3.2.1.3	44	3569	9	730	45	1.36.1.2	29	3477	7	839
19	3.2.1.4	46	3732	7	567	46	3.32.1.8	40	3374	11	927
20	3.2.1.5	46	3732	7	567	47	1.36.1.5	10	1199	26	3117
21	3.2.1.6	48	3894	5	405	48	7.41.5.1	10	2241	9	2016
22	2.28.1.1	18	1246	44	3044	49	7.41.5.2	10	2241	9	2016
23	3.3.1.2	22	3280	7	1043	50	1.41.1.2	36	3692	6	615
24	3.2.1.7	48	3894	5	405	51	2.5.1.1	13	2345	11	1983
25	2.28.1.3	56	3875	6	415	52	2.5.1.3	14	2525	10	1803
26	3.3.1.5	13	1938	16	2385	53	1.41.1.5	17	1744	25	2563

Experiments presented in [8] and associated to 54 protein families of SCOP version 1.53, here ordered by progressive number. For each target family ID is detailed the number of positive and negative sequences of training and test.

For the comparison against state-of-the-art methods we use as metric the receiver operating characteristic (ROC) score. Given a ranking of the test sequences scores in output from the SVM, ROC score is the normalized area under the curve that plots the number of positive examples correctly predicted (true positives) as a function of the number of negative examples found to be positive (false positives) for each different possible classification threshold of a specific experiment. Our results were obtained using the Gist SVM version 2.3; realized by Noble and Pavlidis [30], it implements the soft margin optimization algorithm described in [31].

For every target family we have to extract the irredundant common patterns, compute the score matrix, train the SVM, and then classify the test sequences. The requirements in terms of time can vary dramatically. In general, they are related to the number of sequences and to the protein family. The most time consuming step is the training of the SVM with times that range between few minutes up to 50 minutes, in some difficult cases. Experimental data and results of other methods were taken from [11,20]. Table 5 shows the scores of ROC, ROC50, and Median rate of false positives (mRFP) averaged out over all the experiments for Irredundant Class and state-of-the-art methods. We report in bold the best results for each score. These scores indicate that our method outperforms most learning algorithms in literature, and it is comparable to state-of-the-art Local Alignment kernels.

**Table 5:** Comparison of results against state-of-the-art methods

Learning algorithm	Mean ROC	Mean ROC50	Mean mRFP
Irredundant Class	**0.929**	0.524	0.0554
Local Alignment ("ekm," *β *= 0.5)	**0.929**	0.600	**0.0515**
Local Alignment ("eig," *β *= 0.5)	0.925	**0.649**	0.0541
Word Correlation Matrices (*k *= 6)	0.904	0.447	0.0778
Pairwise	0.896	0.464	0.0837
Mismatch (*k *= 5, *m *= 1)	0.872	0.400	0.0837
Spectrum (*k *= 3)	0.824	0.294	0.1535
Fisher	0.773	0.250	0.2040

The comparison is based on mean scores of ROC, ROC50 (ROC curve up to the first 50 false positives), and Median rate of false positives (mRFP) for the Irredundant Class and state-of-the-art methods. In bold are reported the best results for each score.

**Figure 1:**
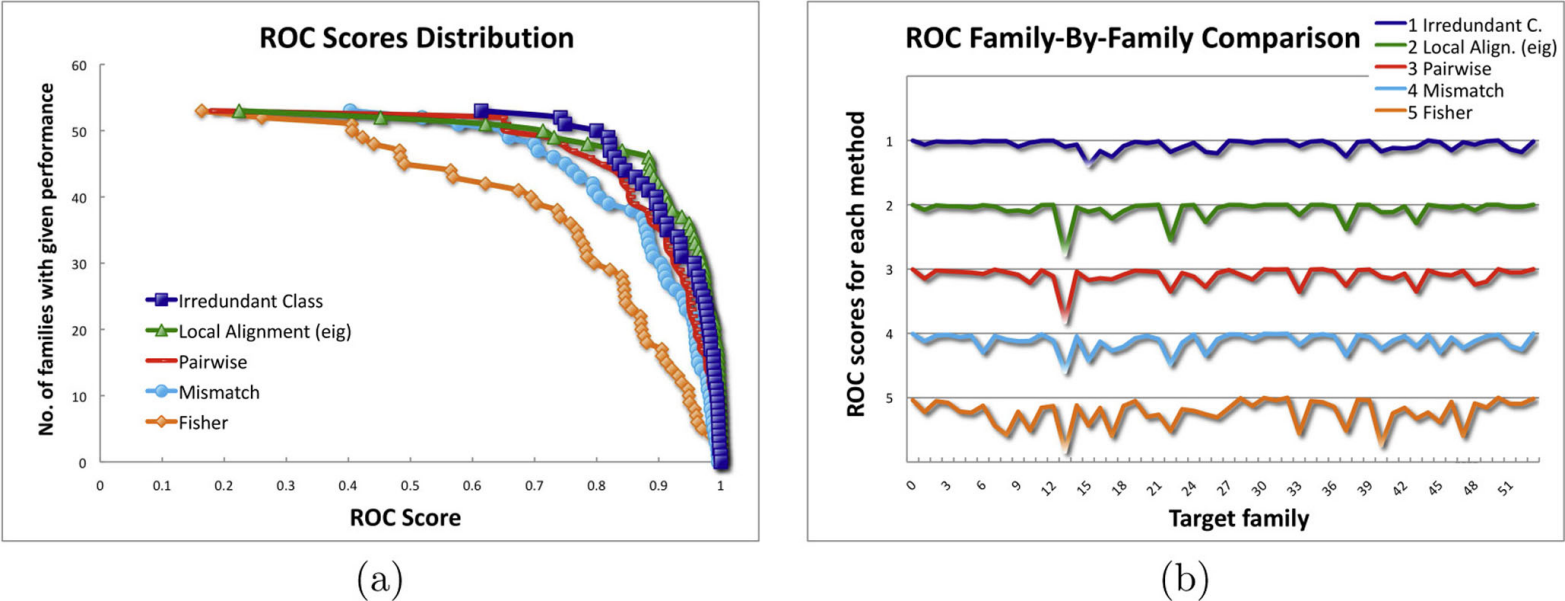
**ROC scores distributions**. (a) ROC scores distribution for the Irredundant Class and state-of-the-art methods. (b) ROC scores across families.

**Figure 2:**
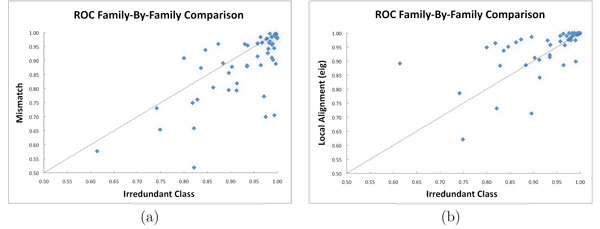
**ROC scores family-by-family comparisons**. (a) Family-by-family ROC scores comparison of the Irredundant Class against Mismatch. (b) Family-by-family ROC scores comparison of the Irredundant Class against Local Alignment version "eig."

For a more detailed view, the ROC scores distribution is illustrated in Figure 1(a). The green line (triangles), corresponding to the Local Alignment version "eig" (that was suggested by the authors for a lower computational expensive with respect to the version "ekm"), seems to perform slightly better than the Irredundant Class in blue (squares), but the minimum ROC score of the Local Alignment is much smaller. Figure 1(b) reports the distribution of ROC scores per family. The Irredundant Class shows a more robust behavior for all experiments with respect to the other learning algorithms. In particular, the smallest ROC score of our method was obtained for the 15-th experiment with the value 0.614, while all other methods get their lowest peaks for the 13-th experiment with values much smaller, e.g. 0.22 for Local Alignment. Family-by-family details can be caught from Figure 2 for the comparison of the Irredundant Class against, respectively, (a) Mismatch and (b) Local Alignment ("eig"). The former is one of the most efficient kernels in literature, while the latter is known to achieve high performance. Again, we can observe that Irredundant Class achieves better performance than Mismatch and similar results when compared with Local Alignment.

### Analysis of irredundant patterns footprint

Although the classification of protein sequences, through an SVM, does not provide an alignment per se, one can use the footprint of irredundant patterns to detect candidate functional sites in protein sequences. Here we are not interested in aligning a set of sequences, but we want to analyze the distribution of the most discriminative irredundant common patterns.

We recall that the result of the SVM learning process, for a target protein family, is a set of weights *α *= (*α*_1_, ..., *α*_*N *_) associated to the *N *training sequences of its superfamily. We want to study the distribution of irredundant common patterns in the test sequences using for each pattern *p *a weight that is proportional to its score  and to the weight *α*_*i *_of the corresponding training sequence that generated *p*, with 1 ≤ *i *≤ *N*. Consider a test sequence *s*_*test *_and the set of training sequences *s*_1_, ..., *s*_*N*_; each pair (*s*_*test*_, *s*_*i*_) generates a set of irredundant patterns ℐ_*test, i*_. For each pattern *p *in ℐ_*test, i *_we compute its score as the product ln () × *α*_*i *_and we associate this score to the positions of *stest *covered by solid characters of *p*. We repeat the same process for all patterns in ℐ_*test, i*_, for each 1 ≤ *i *≤ *N*; and for each location we sum the contributions of all patterns that cover that location. We obtain a histogram of the footprint of the irredundant patterns for the test sequence *s*_*test*_. The interpretation of this histogram is that picks should correspond to conserved regions, thus to candidate functional sites.

**Figure 3:**
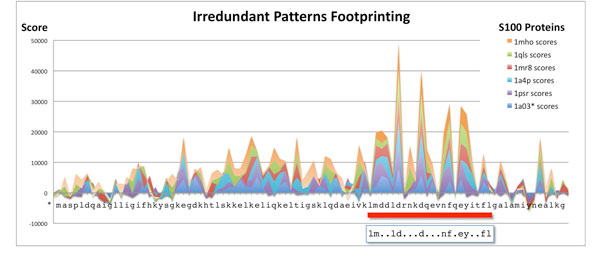
**Irredundant patterns footprint for protein family 50**. Histogram of the irredundant patterns footprint for S100 proteins (family no. 50 of Table 4).

**Figure 4:**
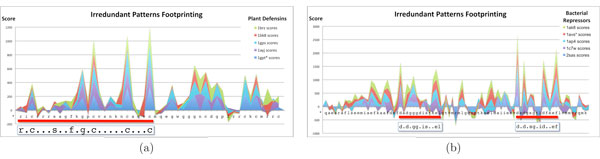
**Irredundant patterns footprint for protein families 32 and 53**. Histogram of the irredundant patterns footprint for: (a) plant defensis (family no. 32 of Table 4) and (b) bacterial repressors (family no. 53).

We picked three families at random from the dataset used. For every protein family we use as target functional sites the PROSITE [26] manually found consensus patterns. To better highlight the distribution of footprints we build, for each family, a multiple alignment of the test sequences and plot all histograms over this alignment. Figure 3 shows the resulting histogram for the protein family S100. A red bar shows the location of the functional pattern reported by PROSITE, also shown in the picture. For this family we can see that a clear signal is present and that picks correspond quite well with conserved amino acids in the functional site. Similar considerations apply also for the families in Figure 4. In Figure 4(a) we observe picks mostly in correspondence of Cysteines, whereas in Figure 4(b) the pattern reported by PROSITE results in two functional sites that share comparable high scores.

Note that these results are obtained comparing sequences from a protein family and its superfamily, thus the chances to find the actual signal are weak as opposed to standard alignment methods, which consider only the protein family. Nevertheless the profile of the family functional site can be computed as a post process of our analysis by a multiple alignment of candidate regions.

This analysis does not yield to an alignment of sequences, but it is a way to interpret the distribution of irredundant patterns. In summary the most discriminative patterns contain information about the functional site of a protein family, but this information is not explicitly available by simple inspection.

## Conclusion

In this paper we studied how the notion of irredundant patterns can solve an issue that is rising in the field of string-based learning algorithms. Almost all string-based approaches consider patterns that are not independent, and therefore the associated scores overcount, a multiple number of times, the contribution of patterns that cover the same region of a sequence, called redundant patterns. Thus we use the class of irredundant common patterns over two sequences, which is specifically designed to address this issue. We design a novel method, called Irredundant Class, that is a discriminative pairwise learning algorithm based on support vector machines. Results on benchmark data show that the Irredundant Class outperforms most of the approaches previously proposed, and it achieves results as good as current state-of-the-art methods. Moreover the footprint of the most discriminative irredundant patterns can be interpreted, in a biological fashion, as a guide for the identification of characteristic regions in protein sequences. Finally, the Irredundant Class approach is not limited to protein sequences, but it can also be applied to DNA or RNA sequences. The investigation of these areas will be part of future work.

## Competing interests

The authors declare that they have no competing interests.

## Authors' contributions

All authors have equally contributed to, seen and approved the manuscript.
